# Hematological parameters in the early phase of influenza A virus infection in differentially susceptible inbred mouse strains

**DOI:** 10.1186/s13104-015-1195-8

**Published:** 2015-06-06

**Authors:** Matthias Preusse, Klaus Schughart, Esther Wilk, Frank Klawonn, Frank Pessler

**Affiliations:** Institute for Experimental Infection Research, TWINCORE Centre for Experimental and Clinical Infection Research, Feodor-Lynen-Str. 7, 30625 Hannover, Germany; Bioinformatics, Helmholtz Centre for Infection Research, Brunswick, Germany; Department of Infection Genetics, Helmholtz Centre for Infection Research, Brunswick, Germany; Helmholtz Centre for Infection Research, Inhoffenstraße 7, 38124 Brunswick, Germany; University of Veterinary Medicine, Hannover, Germany; University of Tennessee Health Science Center, Memphis, TN USA

**Keywords:** Anesthesia, Blood, Influenza A virus, Intranasal infection, Leukocytes, Lymphocytes, Mock treatment, Mouse model, Thrombocytes

## Abstract

**Background:**

Hematological parameters have not received much attention in small animal models of infection, particularly at very early time points. We therefore studied changes in leukocyte and thrombocyte numbers in a mouse model of influenza A virus (IAV) infection, including measurements within the first 24 h after infection, and also assessing effects, if any, of the infection/anesthesia procedure on these parameters.

**Methods:**

DBA/2J and C57BL/6J mice (n = 5–8 per observation) were evaluated in a time course experiment of IAV infection, focusing on early time points. After anesthesia with ketamine/xylazine, a suspension of 2 × 10^3^ focus forming units of the mouse-adapted IAV strain A/Puerto Rico/8/1934 (H1N1) in 20 µl sterile PBS, or 20 µl sterile PBS only (“mock treatment”), were instilled intranasally. Weight loss was assessed daily, and eight common hematological parameters and viral hemagglutinin (HA) mRNA expression were determined after 6, 12, 18, 24, 48 and 120 h.

**Results:**

Hematological differences between the strains were apparent even in untreated mice. Infection-dependent changes, in particular increased granulocyte and decreased lymphocyte counts, were first detectable after 18 h in DBA/2J, were fully manifest in both strains at 48 h, and were usually more pronounced in the DBA/2J mice. In this strain, relative granulocyte and lymphocyte counts and the granulocyte/lymphocyte ratio correlated with viral HA mRNA expression and weight loss. In C57BL/6J, hematological parameters did not correlate with weight loss, but HA mRNA expression correlated weakly with total leukocyte counts, granulocyte/lymphocyte ratio, relative and absolute granulocyte counts, and relative lymphocyte counts. Significant changes due to mock treatment were mild and were detected only in C57BL/6J.

**Conclusion:**

This study underscores the value of hematological parameters in monitoring disease evolution in the early phase of IAV infection, and likely other pathogens. The hematological response to infection may differ significantly among inbred mouse strains.

## Background

Hematological parameters are routinely assessed when evaluating infections in humans. Surprisingly, they are determined infrequently in small animal models of infectious diseases (e.g. [1]). Influenza A virus (IAV) infection represents a common acute infection with a great disease burden in both human and veterinary medicine. The mouse represents a well-established small animal model of susceptibility to IAV infection in an adaptive host [2–4]. For instance, DBA/2J mice have been shown to be highly susceptible to the mouse-adapted IAV strain PR8 (A/Puerto Rico/8/1934 [H1N1]), whereas C57BL/6J mice are relatively resistant, as evidenced by less weight loss, less severe lung pathology, and a lower viral load. DBA/2J mice also mount a hyper-inflammatory response with much stronger up-regulation of many immune response-dependent genes. As exemplified by the aforementioned studies, work in murine models of IAV infection have concentrated on mRNA expression studies, and common peripheral blood cells have been assessed only rarely [5–7]. In a separate study, we have recently assessed immunological and hematological parameters as indicators for disease severity in inbred mice infected with IAV strains of differential virulence [8]. We found that, among the 9 hematological indices assessed, the granulocyte/lymphocyte ratio correlated best with disease severity independent of the mouse or virus strain used. However, in that study we did not evaluate very early time points or any effects due to the anesthesia/infection procedure. We therefore conducted the present study in order to characterize changes in hematological parameters in the very early phase of IAV infection and to test whether any effects due to anesthesia and intranasal installation of buffer only (“mock treatment”) occur in this early time window. We find that significant changes in hematological parameters are first detected 18 h post infection (hpi) in DBA/2J and by 24 hpi in C57BL/6J mice, and are fully manifest in both strains by 48 hpi. Changes are consistently more pronounced in the more susceptible DBA/2J strain, whereas a procedure-related (mock treatment) effect, albeit mild, is observed in the C57BL/6J strain only.

## Methods

### Animal procedures

Female 12–13-week old DBA/2J and C57BL/6J mice (n = 5–8 per time point and treatment) and the mouse-adapted IAV strain PR8 (A/Puerto Rico/8/1934 [H1N1], Institute of Molecular Virology, University of Muenster, Germany; previously described in [4] and [9]) were used. All procedures were carried out essentially as described previously [10]. Briefly, mice were housed under pathogen-free conditions, 5 animals per cage, making sure to keep IAV infected, mock treated, and control mice in separate cages. Mice were anesthetized with ketamine and xylazine. Intranasal infection was carried out using 2 × 10^3^ focus forming units (ffu) of IAV in 20 μl sterile PBS. Mock treatment was identical to real anesthesia/infections except that vehicle only (sterile PBS), not containing virus, was used for intranasal instillation. Mice were weighed on day 0 just before induction of anesthesia and then once daily. Mice were killed by CO_2_ asphyxiation at 6, 12, 18, 24, 48, and 120 h after infection or mock treatment. Untreated mice were used as t = 0 h control. RNA extracted from lung homogenates was used to measure expression of IAV HA mRNA expression. The same samples were used to measure various host-encoded transcripts, the results of which have been published separately [10]. The presented time course experiment served as an extension of the study published in [8] and was thus performed only once.

### Ethics statement

All animal work was conducted according to the national guidelines of the animal welfare law in the Federal Republic of Germany and approved by the local regulatory authority (Niedersächsisches Landesamt für Verbraucherschutz und Lebensmittelsicherheit [LAVES], Oldenburg, Germany; permit number: 33.9.42502-04-051/09).

### Hematological measurements

Whole blood was obtained immediately after CO_2_ asphyxiation by cardiac puncture and collected in tubes containing EDTA as anticoagulant. Blood samples (50 μl) were analyzed with a VetScan HM5™ (Abaxis, Union City, CA, USA) hematology analyzer. The following eight parameters were measured (units in brackets): white blood cell count (WBC [10^9^/L]), absolute counts and percentages of lymphocytes (LYM [10^9^/L]; LYM% [%]), monocytes (MON [10^9^/L]; MON% [%]), and granulocytes (GRA [10^9^/L]; GRA% [%]), and absolute platelet count (PLT [10^9^/L]). The GRA/LYM ratio was calculated.

### Data analysis

Data were analyzed using the R environment and programming code [11]. Outliers in pulmonary expression of viral HA mRNA expression were excluded as described previously [10]. Differences (fold change, FC) were calculated by dividing the mean value of each condition by the mean value of the comparator or control. Analysis of variance (ANOVA) was used to test for trends throughout the time series, adjusting p values for false discovery rate (FDR). Tukey’s Honest Significant Differences Test for homogeneous variances was used for pairwise comparisons. Levene’s test was used to test variance equality. We used a significance threshold of p ≤ 0.05. We used the R function power.anova.test with power and p value thresholds of 0.8 and 0.05, respectively, for post hoc sample size estimation.

## Results

### Kinetics of weight loss and IAV HA mRNA expression

**Figure 1:**
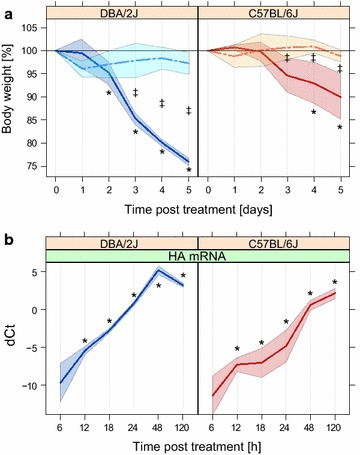
Body weight and HA mRNA expression of mock-treated and infected DBA/2J and C57BL/6J mice. **a** Weight loss. Mice were weighed daily. **b** HA mRNA expression, measured by qRT-PCR in whole lung homogenate as outlined in “Methods”. *Solid lines* infection; *dotted line* mock treatment, *colored area around line* standard deviation. *p ≤ 0.05 compared to untreated mice (weight loss) or compared to 6 hpi (HA mRNA); ^‡^p ≤ 0.05 for difference between infected and mock treated mice at the given time point. All p values were determined with Tukey’s test. This figure is adapted from Ref. [10] and is shown here for the purpose of clarity.

As shown previously [4, 10], DBA/2J mice experienced significant weight loss starting on day 2 and reached a maximum of about 25% by day 5 (Figure 1). Weight loss was less pronounced in C57BL/6J mice, as reflected by a 5–10% decrease starting by day 4. Between days 1 and 5, no significant weight loss due to mock treatment was observed in either strain. HA mRNA was detected as early as 6 hpi, and differences between the mouse strains were already significant at 12 hpi. Overall, HA mRNA rose more rapidly and peaked earlier in DBA/2J than in C57BL/6J.

### Regulation of hematological parameters by infection and mock treatment

We measured absolute white blood cell (WBC) and platelet (PLT) counts, as well as absolute and relative granulocyte (GRA, GRA%), monocyte (MON, MON%), and lymphocyte (LYM, LYM%) counts. Differences between DBA/2J and C57BL/6J mice were clearly evident even in untreated mice in that GRA and GRA% were higher and LYM% was lower in DBA/2J, and the GRA/LYM ratio was substantially higher in DBA/2J (Table 1).

**Table 1:** Selected hematological parameters in untreated mice

Hematological parameter	DBA/2J (mean)	C57BL/6J (mean)	Ratio DBA/2J vs C57BL/6J
WBC	7.1	8.0	0.88
LYM	5.3	6.9	0.76
MON	0.22	0.15	1.5
GRA	1.6	0.94	1.7*
LYM%	74.5	86.6	0.86***
MON%	3.1	1.9	1.6
GRA%	22.4	11.5	1.9***
PLT	664.3	742.2	0.90
GRA/LYM	0.3	0.14	2.2***

Values represent absolute (10^9^/L) and relative (%) mean blood counts.

P values (FDR adjusted): *** ≤0.001 and * ≤0.05.

**Figure 2:**
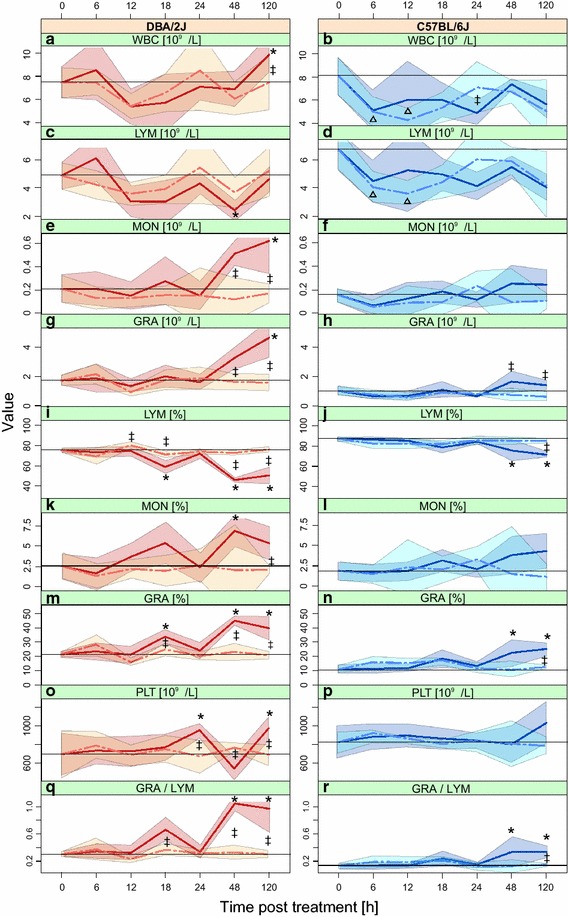
Changes in nine hematological parameters in mock-treated and infected DBA/2J and C57BL/6J mice. Data points were collected across the 5-day time course of IAV infection. *Panels* show mean values and variation of the data (standard deviation) of WBC (**a**, **b**), LYM (**c**, **d**), MON (**e**, **f**), GRA (**g**, **h**), LYM% (**i**, **j**), MON% (**k**, **l**), GRA% (**m**, **n**), PLT (**o**, **p**), and GRA/LYM ratio (**q**, **r**). *Horizontal black lines* indicate values of untreated mice (t = 0 h). * Left panel* results obtained with the DBA/2J mouse strain. *Right panel* results obtained with the C57BL/6J mouse strain. *Solid lines* mice infected with 2 × 10^3^ ffu IAV PR8 M in 20 µl sterile PBS. *Interrupted lines* mice undergoing the same anesthesia/infection procedure except that buffer only, not containing IAV, was used for the intranasal installation (“mock treatment”). *p ≤ 0.05 for difference between infected mice at the given time point with respect to 0 h; ^∆^p ≤ 0.05 for difference between mock treated and control (0 h) mice; ^‡^p ≤ 0.05 for difference between infected and mock treated mice at the given time point. All p values were determined with Tukey’s test.

In infected DBA/2J mice, all eight tested parameters were regulated significantly across the time course (ANOVA, p ≤ 0.05) (Figure 2). In paired comparisons with 0 h, all parameters differed from 0 h at least at one time point (Tukey’s test, p ≤ 0.05). All parameters, except WBC and PLT, changed ≥1.5-fold. None of these parameters changed significantly after mock treatment.

In the C57BL/6J strain, only three parameters (GRA, GRA%, LYM%) changed significantly during infection (ANOVA, p ≤ 0.05), two of which (GRA%, LYM%) differed in at least one time point from 0 h (Tukey’s test, p ≤ 0.05), but the fold change (FC) of LYM% was <1.5. Significant changes were detected in DBA/2J at much earlier time points: an increase in GRA% and a decrease in LYM% were detected at 18 hpi, whereas any changes in C57BL/6J did not become significant until 48 hpi. The GRA/LYM ratio increased in both mouse strains from 48 h onward, with markedly higher ratios being observed in the DBA/2J strain. Thus, infection-related changes in the leukocyte parameters were more time sensitive and pronounced in the DBA/2J strain. Mock treatment-associated changes were exclusively detected in C57BL/6J mice and only affected WBC and LYM. These changes were relatively mild and were limited to the 6 and 12 h time points, but were significant according to both ANOVA and Tukey’s test (FC ≥1.5).

### Sample size estimation

We then performed power calculations to estimate the number of mice needed to achieve statistical significance to detect any regulation (at any time point) of each of the eight measured parameters and the GRA/LYM ratio across the time course. Results are summarized in Table 2. This analysis confirmed that the number of mice used was sufficient to detect infection-related changes in DBA/2J, but that up to 39 mice would be needed to detect additional mock treatment-related effects. For C57BL/6J mice, we estimated that about 10 infected mice (for WBC, LYM, MON, MON% and PLT) and 13 mock treated mice (all parameters except WBC and LYM) would be needed. Thus, more DBA/2J mice would be needed to detect any effects of mock treatment, but more C57BL/6J mice would be needed to detect additional infection-related effects.

**Table 2:** Sample size estimation

Parameter	DBA/2J		C57BL/6J	
	Infection	Mock	Infection	Mock
WBC	3	9	8	4
LYM	4	10	9	4
MON	4	39	11	12
GRA	3	7	4	19
LYM%	2	5	3	8
MON%	4	27	10	16
GRA%	2	7	3	9
PLT	3	9	12	17
GR/LYM	2	6	4	9

Values correspond to numbers of mice needed to detect a statistically significant difference across the time course (power = 0.8, p = 0.05, ANOVA). Values were rounded to the nearest higher integer.

### Correlations with HA mRNA and weight loss

We then tested for correlations of the hematological parameters with measures of viral replication (expression of mRNA encoding the IAV HA protein) and clinical severity of the infection (weight loss). The GRA/LYM ratio, LYM%, and GRA% correlated significantly with HA mRNA expression in both mouse strains (Table 3). MON, MON%, and LYM correlated with HA mRNA expression in DBA/2J mice only. GRA and WBC correlated with HA mRNA expression in C57BL/6J mice only. GRA, GRA%, LYM%, and the GRA/LYM ratio correlated significantly with weight loss in DBA/2J mice. No significant correlation of any parameter with weight loss was observed in C57BL/6J mice.

**Table 3:** Correlations of nine hematological parameters with HA mRNA expression and body weight

Hematological parameter	HA mRNA		Body weight	
	DBA/2J	C57BL/6J	DBA/2J	C57BL/6J
GRA/LYM	0.60***	0.37*	−0.63**	−0.24
GRA%	0.59***	0.37*	−0.64**	−0.24
MON%	0.48**	0.18	−0.29	−0.29
MON	0.44*	0.18	−0.41	−0.19
GRA	0.35	0.43*	−0.58*	−0.02
PLT	−0.11	−0.13	−0.32	−0.06
WBC	−0.16	0.35*	−0.35	0.35
LYM	−0.46*	0.25	0.13	0.40
LYM%	−0.61***	−0.41*	0.64**	0.28

Values correspond to Spearman correlation coefficient in infected mouse strains, sorted by decreasing values in correlation with HA mRNA in DBA/2J mice. HA mRNA expression was measured with real-time RT-PCR in RNA isolated from lung homogenates. P values (FDR adjusted): * ≤0.05, ** ≤0.01, *** ≤0.001.

### Host strain-specific temporal evolution of the hematological response to IAV infection

**Figure 3:**
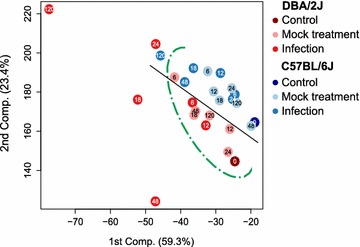
Principal component analysis of eight hematological parameters across the 5-day time course of IAV infection. The parameters WBC, LYM, MON, GRA, LYM%, MON%, GRA%, and PLT were measured in mock-treated and infected DBA/2J and C57BL/6J mice at 6, 12, 18, 24, 48, and 120 h, as well as in untreated mice (0 h). The analysis is based on the same data set as used for Figure 2. *Each dot* refers to the mean value of mice of one treatment as outlined in the legend adjacent to the box. The *number* inside each dot identifies the time (h) elapsed since 0 h. Including the GRA/LYM ratio or the third component did not improve discrimination (data not shown).

In a principal component analysis (PCA, Figure 3) based on the eight parameters (excluding the GRA/LYM ratio), time points and treatments tended to cluster according to mouse strain (black line) and infection status (green curve). As illustrated by the green curve, a cluster containing infected and mock treated time points could be identified easily in both mouse strains. Infected DBA/2J mice clearly segregated from this cluster at 18 hpi, whereas C57BL/6J mice did so much later (120 hpi) and to a markedly lesser extent. These results further support the notion that the overall hematological response to IAV infection was both more sensitive and pronounced in the DBA/2J strain.

## Discussion

This detailed analysis of peripheral blood cell indices in the first 120 h of IAV infection in mice extends previous studies in that we included time points within the first 24 hpi and also assessed any effects due to the anesthesia/infection procedure. This analysis revealed that first significant infection-dependent changes are detected first in DBA/2J (18 hpi) and that procedure-dependent effects are detectable only in C57BL/6J mice; furthermore, it underscored the importance of the GRA/LYM ratio as a sensor of IAV infection.

### Differences between the mouse strains

The response to the infection in the DBA/2J strain evolved both sooner (18 hpi) and more briskly than in the C57BL/6J strain, in which first significant changes were observed not before 48 hpi, and the PCA revealed a global hematological reprogramming even later, i.e. at 120 hpi. What might account for these differences? Firstly, they might be due to differences in viral replication, as the DBA/2J strain is well known to be more permissive to IAV [4], which was confirmed by the more rapid and higher rise in HA mRNA levels in the present study (Figure 1). In this model, the hematological changes would be driven primarily by the pathogen. On the other hand, the differences could also be explained by an inherently higher inflammatory propensity of the DBA/2J strain, which would fit our observation that GRA and GRA% values as well as the GRA/LYM ratio were higher in this strain even at baseline, and that some parameters such as MON and MON% changed in the DBA/2J strain only, despite clear evidence of IAV HA mRNA transcription in both host strains. We favor a combination of both models. LYM% correlated most negatively with HA mRNA expression in both mouse strains, indicating the presence of some common features between the two mouse strains. We could not find a clear regulatory trend in PLT, but this may have been due to a technical error (for instance due to clot formation in samples at this time point) in the PLT measurements at 48 hpi, which showed a lack of change even though the elevation at 24 and 120 hpi would imply an elevation at 48 hpi, too. However, in agreement with these negative findings, our previous study also did not reveal any association of PLT with disease severity in IAV infection in mice [8]. In contrast, the GRA/LYM ratio clearly correlated with disease severity and differed greatly between the two mouse strains even at baseline. This is consistent with previous knowledge that hematological parameters may differ among inbred mouse strains [12] and lends further support to the previously advanced notion that the GRA/LYM ratio is a reliable hematological marker for IAV infection in mice [8].

### Procedure-related effects

A hematological response to mock treatment was not detected in the DBA/2J strain. This was a surprising finding, since cytokine mRNA expression in the lung of DBA/2J mice shows a clear response to mock treatment and, in addition, is stronger than in the C57BL/6J strain [10]. The only observed effect due to mock treatment was a transient elevation in absolute leukocyte parameters in the C57BL/6J strain before 24 h. While there is no obvious explanation for the greater propensity of the C57BL/6J strain for procedure-related artefacts, it is conceivable that some links between airway stress and the immune response may be more reactive in this strain.

### The value of hematological parameters as biomarkers of IAV infection in mice

The presented study sheds additional light on the value of using hematological parameters to follow the evolution of early IAV infection in mice. Traditionally, peripheral blood cytokines/chemokines or flow cytometric analyses of peripheral leukocyte populations have been used to follow the host response to viral infections in the periphery, whereas the use of common hematological parameters (a much more cost- and labor-efficient read out) has not received much attention in research settings. This is all the more surprising as highly standardizable, easy-to-use equipment and well validated reference values for many parameters are available. The most reliable infection-related changes in both mouse strains were observed in LYM% and GRA% at a time when the infection was relatively well established (48 hpi), with the GRA/LYM ratio correlating best with pulmonary HA mRNA expression when considering both mouse strains. However, the first significant changes were detected as early as 18 hpi in the DBA/2J strain. This raises the possibility that certain leukocyte populations in peripheral blood might serve as surrogate markers for early establishment and subsequent progression of influenza infection in mice. While hematological parameters in established IAV infection in mice have been studied in sufficient cumulative numbers of mice [5–8], further studies featuring larger sample sizes and additional time points will be necessary to understand the value of these parameters within the first 24 h better. Our results agree well with the findings of our recent study [8] in which peripheral blood indices were measured daily from 24 hpi until 14 days pi. That study demonstrated that the GRA/LYM ratio increased significantly as early as day 2 pi and stayed elevated through day 5. This ratio also correlated with disease severity, for instance when results were obtained from mice infected with IAV strains of different virulence or when the more susceptible DBA/2J and the C57BL/6J host strain were infected with the same IAV strain [8]. Taken together, these results suggest that hematological parameters (especially GRA/LYM and LYM%) can be used as robust biomarkers for progression of IAV infection starting at 48 hpi, whereas their use at earlier time points may depend more on the mouse strain used and will certainly benefit from additional validation. The increased GRA/LYM and decreased LYM% clearly resulted from rising GRA counts accompanied by dropping LYM counts. Both granulocytosis (as part of the acute phase reaction) and lymphopenia have been well documented in viral pneumonia in humans (e.g. [13]). The temporary drop in lymphocytes, which is the stronger determinant of the early changes in GRA/LYM and LYM% in both mouse strains (Figure 2) might be due to migration/retention of lymphocytes in lung and/or lymphoid tissue.

### Value of the sample size calculations

For ethical and humanitarian reasons it is important to use the lowest number of laboratory animals needed to achieve statistical power. The analysis clearly showed that fewer DBA/2J than C57BL/6J mice were required to achieve statistical significance, suggesting that—assuming infection with equal infectious doses and viral strains—smaller numbers of animals need to be used when working with the DBA/2J strain, thus allowing more efficient planning of experiments and using the optimal number of animals depending on the mouse strain to be used. Interestingly, it appears that even in the C57BL/6J strain, using approx. 10–12 animals per time point might suffice to study most infection-related changes in hematological parameters, a number that is similar to what is often used in mouse models of infectious diseases.

## Conclusions

This study underscores the value of using hematological parameters to track the evolution of IAV infection in mice, even in very early phases of infection. It also suggests that “mock treatment” controls should be included in order to exclude any artefacts, albeit mild, arising from the infection/anesthesia procedure.

## Abbreviations

ffu: focus forming units
GRA: absolute granulocyte count
GRA%: percentage of granulocytes
h: hour
HA: hemagglutinin
IAV: influenza A virus
LYM: absolute lymphocyte count
LYM%: percentage of lymphocytes
MON: absolute monocyte count
MON%: percentage of monocytes
pi: post infection
PLT: absolute platelet counts
WBC: absolute white blood cell count
